# Network physiology in space: vision and perspectives on exploring physiological networks during spaceflight for the benefit of life on earth

**DOI:** 10.3389/fnetp.2026.1817815

**Published:** 2026-04-24

**Authors:** Nandu Goswami, Jerry Joseph Batzel, Yaopeng J. X. Ma, Per Morten Fredriksen, David Andrew Green, Plamen Ch. Ivanov

**Affiliations:** 1 Gravitational Physiology and Medicine Research Unit, Division of Physiology and Pathophysiology, Otto Loewi Research Center of Vascular Biology, Immunity and Inflammation, Medical University of Graz, Graz, Austria; 2 Center for Space and Aviation Health, Mohammed Bin Rashid University of Medicine and Health Sciences, Dubai, United Arab Emirates; 3 Department of Mathematics, University of Graz, Graz, Austria; 4 Keck Laboratory for Network Physiology, Department of Physics, Boston University, Boston, MA, United States; 5 Faculty of Social and Health Sciences, Department of Public Health and Sport Sciences, Section for Public Health, University of Inland Norway, Innlandet, Norway; 6 Space Medicine Team, European Astronaut Centre, European Space Agency, Cologne, Germany; 7 Centre of Human and Applied Physiological Sciences, King’s College London, London, United Kingdom; 8 Institute for Risk and Disaster Reduction, University College London, London, United Kingdom; 9 Department of Neurosurgery, Boston University Chobanian & Avedisian School of Medicine, Boston, MA, United States; 10 Institute of Biophysics and Biomedical Engineering, Bulgarian Academy of Sciences, Sofia, Bulgaria

**Keywords:** artificial intelligence (AI), human physiolome, microgravity, multi-omics, network physiology, physiological deconditioning, spaceflight, systems biology

## Abstract

Network Physiology provides a unifying framework for understanding how molecular, cellular, and organ-level systems integrate as a network to generate distinct physiological states and sustain human function. Spaceflight offers a unique environment—characterized by microgravity, radiation, isolation, and circadian disruption—that perturbs interconnected physiological systems and networks. Network Physiology in Space, an emerging area of research and clinical practice within the multidisciplinary field of Network Physiology, examines how multiscale interactions, from genomic and metabolic pathways to organ system dynamics, adapt and reorganize in response to spaceflight stressors to maintain homeostasis at the organism level. Using systems biology, multi-omics, nonlinear analyses of physiological systems dynamics, computational modeling, and AI-enhanced analysis, researchers have traditionally focused on individual systems to investigate regulatory mechanisms underpinning adaptations to spaceflight, including muscle and bone loss, cardio-vascular and cardio-respiratory deconditioning, immune function shifts, neuro-vestibular dysregulation, circadian, and sleep fragmentation. However, physiological systems and organs continuously interact across levels to synchronize dynamics and coordinate functions. Changes in a system in response to perturbations are often interlinked with other systems, leading to diversity of effects, which underscores the need for an integrative framework capable of linking molecular signals to system-level physiological function and crew functionality. In this context, Network Physiology provides a unifying theoretical and analytical approach to identify, quantify and model dynamic interactions among physiological systems across spatio-temporal scales, integrating multi-omics, physiological, and behavioral data into dynamical network representations. This systems-level perspective enables spaceflight-induced adaptations to be interpreted as coordinated reconfigurations of interacting physiological networks, rather than isolated responses of individual components. As many adaptations are common with disuse pathology, spaceflight becomes a living laboratory for probing frailty and resilience, revealing principles relevant to aging, metabolic and immune disorders, neurodegeneration, and rehabilitation on Earth. Recent methodological advances in inferring functional forms of coupling and causality in dynamic systems interactions, and novel integrative and adaptive network approaches in Network Physiology offer new perspectives to human and animal studies in space or analogue environments, for the development of translational applications to clinical practice and hybrid mechanistic–machine-learning models that simulate system-wide responses and guide personalized countermeasures strategies and personalized medicine.

## Introduction

Despite significant advances in systems biology and integrative physiology over recent decades, a complete understanding of how diverse physiological systems and organs coordinate their dynamics across spatial and temporal scales is currently lacking. Specifically, the mechanisms through which systems integrate both vertically, from genomic, proteomic, and metabolic interactions to cellular signaling pathways, tissues, and organs, and horizontally, as interconnected networks, to generate distinct physiological states, environmental adaptations, maintain hemostasis (health), precipitate disease phenotypes, and influence pathophysiological, symptomatic and functional associations between diseases, remain unclear. Recent developments in coupled nonlinear systems, statistical and computational physics, signal processing, information theory, and adaptive networks of dynamical systems offer new tools to uncover the complexity of physiological systems, their interactions, and their effects. These tools form foundational elements of the theoretical frameworks and methodological approaches to yield a new age of physiology, to inform new dawn in (personalized) medicine.

**FIGURE 1:**
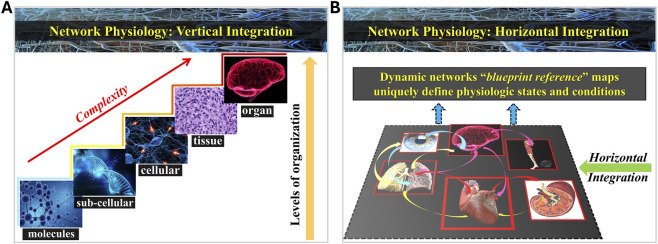
Vertical and horizontal integration of physiological systems. The human organism is an integrated network in which diverse physiological systems continuously interact to coordinate function and maintain health. **(A)** Vertical integration of physiological systems across levels of organization. Current research in Systems Biology and Integrative Physiology has primarily focused on signaling and feedback mechanisms operating across space and time scales—from molecules and sub-cellular processes to cellular, tissue, and organ levels—where complexity progressively increases with higher levels of organization. **(B)** Horizontal integration within levels of physiological organization is essential to facilitate functions at each level and generate distinct states and conditions. While medicine traditionally defines health and disease through the state of individual physiological and organ systems, Network Physiology emphasizes dynamic interactions among systems within and across organizational levels, where coordinated network activity (“blueprint reference” maps) uniquely defines physiological states and conditions. A fundamental question is how physiologic states and functions emerge out of network interactions through vertical and horizontal integration among systems. In the context of space physiology and medicine, Network Physiology investigates how spaceflight conditions affect vertical and horizontal integration, reshaping the dynamics of network interactions across physiological systems from the molecular and omics level to the organism level.

A central challenge in any biological, including physiological systems is quantifying and mechanistically understanding how global behavior emerges from network interactions among dynamically evolving entities, particularly when the coupling between components is time dependent. In the context of systems biology, human physiology, and medicine, this challenge has led to the emergence of the new field of Network Physiology (Bashan et al., 2012; Ivanov and Bartsch, 2014; Ivanov, 2021), propelled by advances in biomedical technologies, computational modeling, and theoretical approaches (Figure 1).

Traditional systems biology, following the reductionist paradigm, has largely focused on identifying key molecular elements within cells and elucidating their roles in cellular function by evaluating the effects of functional knockouts. Integrative network approaches have extended these efforts by constructing knowledge graphs that represents static maps of relationships (statistical associations) among cellular components, aiming to uncover complex intra-cellular signaling pathways and systematically organize genomic, proteomic, and metabolic data. Such approaches have catalyzed a paradigm shift, enabling the identification of associations between clusters of disease phenotypes and shared genes.

Graph theory and complex networks have also been employed to predict the roles of gene and proteins in disease expression based on their network neighborhood, through motifs and modules, which has highlighted novel genes involved in numerous disease phenotypes (Milo et al., 2002; Stuart et al., 2003; Song et al., 2005; Oti et al., 2006; Köhler et al., 2008; Ivanov et al., 2016). This static graph-based perspective has facilitated the identification of many previously unknown disease-associated genes, yet much work remains given that only 10%–15% of all human genes have known disease associations (Amberger et al., 2009; Barabási et al., 2011).

Moreover, integrative frameworks have expanded understanding of intra-cellular networks to encompass tissue- and organ-specific gene connectivity, establishing principles of gene interactions across cells and tissues (Lage et al., 2008; Reverter et al., 2008; Kirouac et al., 2010; Lage et al., 2010). Recent innovations in layered multiplex networks integrate genetic networks with co-morbidity factors, providing insights into how various risk factors influence gene function, and thus, the likelihood of disease expression in complex clinical scenarios (Lee et al., 2008; Park et al., 2009) as well as the temporal evolution of co-morbidity and diseases networks (Haug et al., 2021; Dervić et al., 2025).

While systems biology has primarily addressed vertical integration (Joyner, 2011), from sub-cellular to cellular and tissue levels, its methodological focus has traditionally been on bottom-up mechanistic causation and static cross-scale associations. As a result, important gaps remain both in understanding the dynamic nature of vertical integration across levels, where differences in time scales and governing principles complicate cross-communication, as well as in horizontal integration among interacting physiological and organ systems (Figure 1), where heterogeneous dynamics and regulatory mechanisms coexist within the same level of organization (Bartsch and Ivanov, 2014; Bartsch et al., 2014). These limitations constrain our ability to explain how coherent organism-level behaviors emerge from distributed physiological processes.

Network Physiology (Bashan et al., 2012; Ivanov and Bartsch, 2014; Ivanov et al., 2016; Ivanov et al., 2017; Ivanov, 2021) addresses these challenges by studying how physiological systems coordinate, synchronize, and integrate as a network to optimize functions and maintain health, and the cascade failure may underpin their failure. In this framework, network nodes represent distinct dynamical physiological systems operating at different scales, each with distinct dynamics and control mechanisms, while network links reflect transient dynamical coordination between systems. A central question in Network Physiology is how global physiological states (and adaptations) emerge from the collective dynamics of interconnected organ systems (Bartsch et al., 2015; Liu et al., 2015b). A key challenge is that small temporal variations in individual system dynamics or network interactions appear to lead to markedly different global behaviors, even in networks with unchanged topology (Bashan et al., 2012; Liu et al., 2015a; Lehnertz et al., 2020; Lin et al., 2020; Chen et al., 2022).

In fact, physiological interactions occur across multiple levels and spatio-temporal scales, producing distinct physiological states such as rest and fatigue, wakefulness and sleep, consciousness, and unconsciousness. As such, investigations in Network Physiology focus on: (i) the structural and functional connectivity within individual physiological and organ systems and their sub-systems (Liu et al., 2015a; Garcia-Retortillo and Ivanov, 2022), and (ii) how organism-level behaviors arise from interactions among systems, shaping health or disease outcomes (Bashan et al., 2012; Ivanov and Bartsch, 2014; Rizzo et al., 2020; Günther et al., 2022; Rizzo et al., 2023). Understanding these interactions is critical as disruption of networked communications can not only lead to dysfunction of individual systems, but precipitate rapid systemic collapse, such as coma or multi-organ failure (Buchman, 2006; Moorman et al., 2016; Berner et al., 2022). Consequently, Network Physiology complements traditional approaches by emphasizing the importance of system-wide coordination and hierarchical network integration as hallmarks of physiological state and function (Lin et al., 2016; Ivanov et al., 2021; Rizzo et al., 2022).

## Physiological systems: vertical and horizontal integration from molecules to organ networks

Building on the framework outlined above, physiological systems can be conceptualized along two complementary dimensions: vertical integration, spanning molecular, cellular, tissue, and organ levels; and horizontal integration, representing the dynamic coordination among organs and systems within the body (Figure 1). Considering these dimensions jointly is essential for understanding how homeostasis in health is maintained, and how pathological states emerge, as well as how interventions at one level propagate through the physiological network.

### Vertical integration: from molecules to organs

Vertical integration describes the cascade of biological interactions that originates at the molecular level and manifests as organ-level function. At the molecular level, proteins, nucleic acids, lipids, and small molecules interact within defined pathways to regulate cellular behavior. For example, the binding of insulin to its receptor triggers intracellular signaling through the phosphoinositide 3-kinase (PI3K)/Akt pathway, which modulates glucose uptake and glycogen synthesis in hepatocytes and myocytes (Taniguchi et al., 2006). This molecular signaling underlies the physiological response of glucose homeostasis at the organ and systemic levels.

At the cellular level, vertical integration encompasses how cells coordinate their internal processes and respond to external stimuli. Cellular activities such as metabolism, ion transport, gene expression, and signal transduction collectively define the functional state of tissues. In cardiac tissue, cardiomyocytes exhibit a tightly regulated excitation-contraction coupling mechanism, whereby the molecular dynamics of ion channels and calcium handling proteins generate coordinated contractions across the tissue (Bers, 2002). This cellular orchestration directly underpins the pumping function of the heart, demonstrating a clear link between molecular events and organ-level output.

Tissue-level integration reflects how specialized cell populations organize into functional units. For instance, in the liver, hepatocytes, Kupffer cells, and hepatic stellate cells interact within the lobular architecture to maintain metabolic homeostasis, detoxification, and immune surveillance. Disruption at any cellular or molecular node, such as in non-alcoholic fatty liver disease (NAFLD), propagates through the tissue architecture, altering organ function (Tilg and Moschen, 2010). These examples illustrate that vertical integration is not merely additive; it is a hierarchical networked system where molecular perturbations can generate organ-level phenotypes.

Organ-level function represents the culmination of vertical integration. Physiological outputs, such as cardiac output, renal filtration, or pulmonary gas exchange, emerge from the integrated activity of tissues and cellular networks. Furthermore, these organ-level functions are subject to feedback regulation. The kidneys, for instance, regulate systemic blood pressure through the renin-angiotensin-aldosterone system (RAAS), which is initiated by molecular sensing of perfusion pressure and sodium concentration, and coordinated by cellular and tissue-level responses. This exemplifies how vertical integration ensures that organ function is not an isolated process, but a dynamic product of multiscale interactions.

The multidisciplinary field of Network Physiology provides a unifying theoretical framework for the hierarchical perspective of vertical integration by emphasizing the dynamic coupling among networks operating at different spatial and temporal scales, conceptualizing each level of biological organization—metabolic, genomic, cellular, tissue, and organ—as an inter-connected network embedded within a larger “network of networks.” Vertical integration, in this framework, reflects the coordinated exchange of information and dynamical interactions across levels, whereby changes in one node or network alter the coupling structure and functional organization of others. Thus, physiological states emerge not only from structural hierarchy but from multiscale dynamical coordination, temporal synchronization, and adaptive network reconfiguration among components across layers of biological organization.

### Horizontal integration: inter-organ network dynamics

Horizontal integration refers to the continuously dynamical interplay among physiological and organ systems to achieve systemic homeostasis. Whereas vertical integration concerns interactions across hierarchical levels of biological organization, horizontal integration refers to interactions among systems operating at the same level of organization. Physiological systems in the organism do not act in isolation; instead, physiological states and functions at the organism level emerge from coordinated multi-organ responses. For example, the regulation of blood glucose involves the pancreas (insulin/glucagon secretion), liver (glycogen storage and gluconeogenesis), skeletal muscle (glucose uptake), and adipose tissue (lipid metabolism). Disruption in any node or alteration in inter-system coupling can precipitate systemic dysfunction, as observed in type 2 diabetes mellitus (DeFronzo et al., 2015).

Another canonical example of horizontal integration is the cardiovascular and renal systems. Blood pressure and volume are controlled by a tightly coupled network involving the heart, kidneys, vasculature, and endocrine organs. The heart pumps blood, the kidneys adjust fluid and electrolyte balance, and the vasculature modulates resistance—all coordinated through neural and hormonal signals. This coordination exemplifies network-level dynamics: feedback from one organ alters the function of others through an adaptive network of time-varying “elastic” interactions, and systemic stability emerges from these inter-organ interactions (Ivanov and Bartsch, 2014; Hall, 2016; Ivanov, 2021).

The neuroendocrine system provides another demonstration of horizontal integration. Hypothalamic-pituitary axes regulate diverse physiological processes, including stress response, reproduction, and growth, by coordinating hormone secretion across multiple target organs. The hypothalamic-pituitary-adrenal (HPA) axis, for instance, modulates cortisol secretion, which in turn affects liver metabolism, immune function, and cardiovascular tone (Charmandari et al., 2005; Goswami et al., 2019). Disruption in this network can lead to systemic pathophysiology, emphasizing the integrative nature of organ communication (Voliotis et al., 2018).

Horizontal integration also extends to organ systems that traditionally appear disparate. The gut-brain axis exemplifies a bidirectional network where the microbiome influences central nervous system function via immune, endocrine, and neural pathways. Metabolites such as short-chain fatty acids and neurotransmitter precursors produced by gut microbes modulate brain function, while neural and hormonal outputs from the brain regulate gut motility, secretion, and microbial composition (Cryan and Dinan, 2012; Takayasu et al., 2017). Horizontal integration among key organ systems—brain, cardiac, respiratory, muscular, ocular—is essential in facilitating basic physiological states such as wake and sleep, rest and exercise, where distinct network structure and hierarchy in the strength and directionality of systems interactions uniquely define each physiological state, and where specific pathways of network reorganization underlie transitions across states (Bartsch et al., 2015; Liu et al., 2015b; Ivanov et al., 2021; Garcia-Retortillo and Ivanov, 2022; Rizzo et al., 2023; Garcia-Retortillo and Ivanov, 2025; Ivanov and Bartsch, 2025). This inter-organ communication demonstrates that physiological systems are networked across traditional anatomical boundaries.

### Network physiology: bridging vertical and horizontal dimensions

The true complexity of physiological systems emerges when vertical and horizontal integration are considered jointly across multiple organ systems and time scales (Figure 1). A classic example is exercise physiology. During exercise, skeletal muscle metabolism is modulated at the molecular level by AMP-activated protein kinase (AMPK) and calcium-calmodulin-dependent protein kinase pathways, which enhance glucose uptake and mitochondrial biogenesis (Hardie et al., 2012). Cellular energy consumption triggers tissue-level responses, such as increased capillary perfusion, while organ-level outputs include elevated cardiac output and respiratory ventilation to match, but not exceed metabolic demand. Horizontally, these responses are coordinated across the cardiovascular, respiratory, endocrine, and nervous systems to ensure oxygen delivery, substrate utilization, and thermoregulation (Balagué et al., 2020; Balagué et al., 2022a; Balagué et al., 2022b). This multiscale, multi-organ integration illustrates the inherent sophistication of physiological regulation.

Pathophysiological states further underscore the necessity of concurrent understanding both vertical and horizontal dimensions. Hypertension, for instance, can result from molecular alterations in ion channels, cellular changes in vascular smooth muscle, tissue remodeling in arteries, and systemic dysregulation of inter-organ networks involving the kidneys, vasculature, heart, and central nervous system (Carretero and Oparil, 2000). Effective therapeutic interventions often require targeting multiple levels simultaneously, pharmacologically modulating molecular pathways, improving tissue function, and restoring inter-organ coordination, and thus, functionality and organism homeostasis.

## Current challenges in spaceflight-related biomedical risks

After over seven decades of human spaceflight, our frameworks for understanding and mitigating space-related biomedical risks remain nascent (Goswami et al., 2026a). Traditional space medicine is geared to risk prevention by selecting out risk factors, requiring extensive exercise, whilst providing basic acute care (managing injuries or sudden illness in orbit) with periodic health assessment including physician consultation monitoring “classic” physiological parameters in near-real time. However, space medicine is far from modern precision medicine approaches on Earth that leverage big data, advanced analytical approaches, and population-based genomics, and ultimately assumes medical evacuation is an option.

Despite major advances in biomedical science over the past decades, health and disease are still largely defined in terms of individual molecular pathways, physiological and organ systems, rather than through a holistic understanding of their dynamic coordination and network integration across levels of organization in the human organism. The traditional reductionist paradigm in basic science and medicine limits our ability to capture the regulatory network mechanisms that generate vital physiological states and functions, and sustain physiological homeostasis, stability and adaptability under stress. Moreover, reductionist approaches do not provide the framework necessary to explain how perturbations to a given system or sub-system propagate across systems to produce systemic dysfunctions, cascade of failures and multi-system breakdown. In extreme environments, such as spaceflight, where multiple stressors act simultaneously on diverse systems, the limitations of the reductionist paradigm become particularly consequential. This motivates the development of an integrative Network Physiology framework, to explore the laws of cross-system communication and the principles of hierarchical network integration among diverse systems in the human organism as fundamental determinants of health and disease (Ivanov and Bartsch, 2014; Ivanov et al., 2017; Ivanov, 2021; Ivanov et al., 2021).

As deep-space missions venture beyond Low Earth Orbit, without real-time medical support from Earth, there is a pressing need to transition to Earth-independent healthcare systems and optimized countermeasures (Scott et al., 2020). This means equipping crews with autonomous health monitoring and decision-support tools that can predict, and guide treatment of illness before they become mission-threatening (Goswami et al., 2012; Goswami et al., 2013). Such a paradigm shift in astronaut healthcare will rely on advanced technologies: (i) AI for data interpretation and decision support, and informed multi-omics profiling. However, a key impediment to AI and multi-omic approaches is a lack of systematically acquired data volume that commercial spaceflight may be better able to deliver than traditional ‘professional’ spaceflight (Green, 2024); (ii) Integrated platforms of medical devices and sensor networks of spatially distributed, autonomous devices (nodes) that cooperatively monitor and synchronously record high-frequency multi-channel physiological data in space (Ivanov, 2021). However, even with increasing availability of systematically recorded data, a critical challenge lies in understanding how spaceflight perturbs the dynamic interactions among physiological systems, rather than isolated molecular pathways or organs. Such interactions may underpin the high levels of intra-individual variability seen in physiological adaptation to spaceflight, and even its ground-based analogues such as long duration head down tilt bed rest (Scott et al., 2021). Addressing this challenge requires the development of novel analytic methodologies and an integrative Network Physiology framework, and their application to space physiology and medicine. Within this framework, physiological adaptation and dysfunction are understood as network-level processes, reflecting changes in the structure, dynamics, causality, and integration of interacting systems under conditions in which the combined effects of microgravity, radiation exposure, circadian and metabolic disruption, confinement, and isolation may be active. These approaches will also help address the challenge of relatively low volumes of multi-modal data recorded in space compared to some terrestrial studies.

## Network physiology as a unifying framework for precision space health

Spaceflight challenges human health through the combined action of microgravity, radiation exposure, isolation, confinement, and circadian disruption. However, these stressors do not affect physiological systems independently, but instead also perturb the dynamic interactions and functional coupling among organ systems that collectively sustain global physiological states.

**FIGURE 2:**
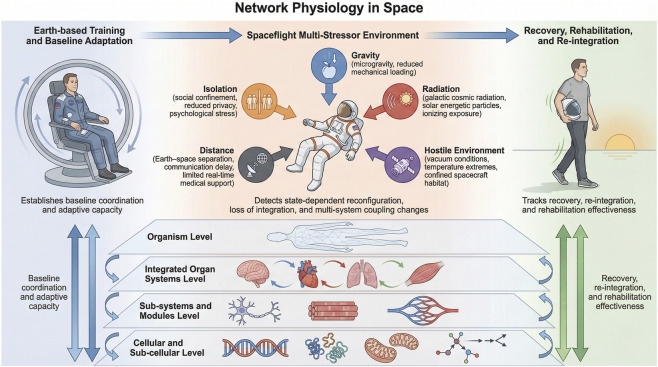
Network Physiology in Space: a multi-scale framework across the spaceflight timeline. Schematic illustration of the Network Physiology framework that integrates multi-scale physiological systems interactions across the spaceflight timeline from Earth-based training to spaceflight and post-flight recovery. Earth-based training establishes baseline coordination and adaptive capacity across physiological systems and networks. During spaceflight, astronauts are exposed to a multi-stressor environment, including altered gravity, high acceleration forces, radiation, hostile conditions, Earth–space distance, confinement, and isolation, leading to state-dependent reconfiguration and altered multi-system interactions. Network Physiology provides methodological and theoretical framework to link cellular, sub-system, organ-system, and organism-level dynamics to capture changes in systems network integration in response to stressors during spaceflight. Following return to Earth, the Network Physiology framework enables tracking of recovery, re-integration, and rehabilitation effectiveness through the re-establishment of cross-scale coordination and network dynamics among systems.

Traditional space medicine has largely focused on isolated molecular, cellular, or organ-level changes (Ivanov et al., 1999; Goswami et al., 2021; Siddiqui et al., 2021; Mason et al., 2024; Bailey et al., 2025), leaving a critical gap in understanding how spaceflight alters the integration, coordination, and adaptability of physiological networks. Network Physiology provides a multidisciplinary theoretical and analytical framework to evaluate these complex systems by explicitly characterizing how physiological systems interact as a coordinated adaptive network across hierarchical levels of physiological organization, from genomic and metabolic networks to organ systems and whole-organism function (Figure 2).

Over the past decade, terrestrial studies in Network Physiology have demonstrated that (i) distinct physiological states are characterized by unique network structure and dynamics of systems interactions, and that (ii) transitions across states and conditions in health and disease are associated with coordinated reconfiguration of cross-system interactions (Bartsch et al., 2015; Liu et al., 2015a; Liu et al., 2015b; Rizzo et al., 2022; Rizzo et al., 2023). Skeletal muscle function, for example, is governed by inter-muscular coordination networks that degrade with fatigue and aging, and reorganize with structured training (Garcia-Retortillo and Ivanov, 2022; Garcia-Retortillo et al., 2023), while maladaptive responses to prolonged stress have been conceptualized as emergent network dysregulation in complex systems such as overtraining syndrome (Romero-Ortuño et al., 2021; Armstrong et al., 2022). Network Physiology of aging research further shows progressive breakdown of inter-organ coordination and resilience (Romero-Ortuño et al., 2021; Garcia-Retortillo et al., 2025b), and restoration of muscle network organization in older adults accompanying functional recovery after targeted intervention (Garcia-Retortillo et al., 2025a). Similarly, physiological stressors related to circadian and sleep disruption were found to reorganize synchronization and functional coupling across behavioral, metabolic, and neurophysiological systems (Healy et al., 2021; Tubbs et al., 2022), and that cardio-respiratory regulation exhibits state- and age-dependent changes in coupling strength and directionality (Bartsch et al., 2012; Borovkova et al., 2022; Abreu et al., 2023). Time-varying information measures further reveal adaptive brain–heart interactions reflecting dynamic autonomic and cognitive states (Antonacci et al., 2023). Although the Network Physiology framework has not been applied to in-flight data yet, the accumulated findings from terrestrial studies collectively establish a conceptual and analytical foundation for investigating how the multi-stressor environment during spaceflight may affect the vertical and horizontal network integration across physiological systems and would be essential to develop more comprehensive strategies for prevention, recovery and treatment of space-related disorders.

The Network Physiology in Space perspective (Figure 2) extends to a mission-phase timeframe investigations encompassing pre-flight, in-flight, and post-flight assessment. Spaceflight provides a dynamic, complex challenge characterized not only by data collection in flight but also pre- and post-flight—termed baseline data collection (BDC). BDC is a key part of any mission not only because comprehensive data collection in flight is challenging or impossible (e.g., MRI), but also because of the need to prepare for spaceflight, and to track and promote re-adaptation and rehabilitation for life back on Earth.

### Earth-based training and baseline adaptation

During Earth-based training and pre-flight preparation, astronauts undergo controlled physiological challenges such as short-arm centrifugation to experience g loading, confinement, altered loading such as acute exposure to microgravity in parabolic flight, and familiarity with inflight exercise paradigms. Network Physiology provides quantitative tools to establish baseline patterns of multi-system coordination, capturing normative interactions among systems including the cardiovascular, respiratory, neuromuscular, and neural systems known to be modulated in space (Goswami et al., 2026a). Previous studies on Earth have shown that physiological network structure and dynamics systematically reorganize across fundamental states such as sleep and wake, rest and fatigue, and aging, offering a reference framework against which spaceflight-induced deviations can be assessed (Figure 2).

### Spaceflight exposure and in-flight adaptation

During spaceflight, exposure to microgravity, radiation, and psychosocial stressors leads to profound alterations in circulation, musculoskeletal loading, neuro-vestibular control, immune regulation, and psychophysiological state (Goswami et al., 2026a). Within the Network Physiology framework, these changes are interpreted as state-dependent reconfigurations of interacting physiological networks, rather than isolated adaptions/dysfunction of individual systems. Studies of brain–heart (Faes et al., 2014; Lin et al., 2016; Valenza et al., 2025), brain–muscular (Rizzo et al., 2020; Roeder et al., 2020; Rizzo et al., 2022; Roeder et al., 2024), carido-respiratory (Bartsch et al., 2012; Abreu et al., 2023), cardio–muscular (Garcia-Retortillo and Ivanov, 2025), and inter-muscular (Kerkman et al., 2018; Garcia-Retortillo and Ivanov, 2022; Garcia-Retortillo et al., 2023) interactions on Earth demonstrate that coupling strength, directionality, and characteristic time delays are sensitive markers of functional state and stress, suggesting that similar network-level signatures may reveal early indicators of maladaptation during spaceflight (Figure 2).

### Return to earth, recovery, and rehabilitation

Following return to Earth, astronauts undergo a prolonged period of physiological recovery and rehabilitation, during which the restoration of ‘normal’ (that is, 1 g appropriate) function depends on the integrated response of multi-system networks. Network Physiology enables longitudinal tracking of how physiological coupling patterns recover, remain altered, or reorganize into compensatory configurations. Evidence from aging, fatigue, and neurodegenerative conditions indicates that incomplete recovery is often associated with persistent breakdowns in network coordination (Rizzo et al., 2023; Garcia-Retortillo et al., 2025a; Garcia-Retortillo et al., 2025b), underscoring the importance of the network-based metrics for evaluating rehabilitation strategies and effectiveness, as well as long-term health risks after spaceflight (Figure 2).

## Methodological approaches to physiological systems integration in spaceflight

**FIGURE 3:**
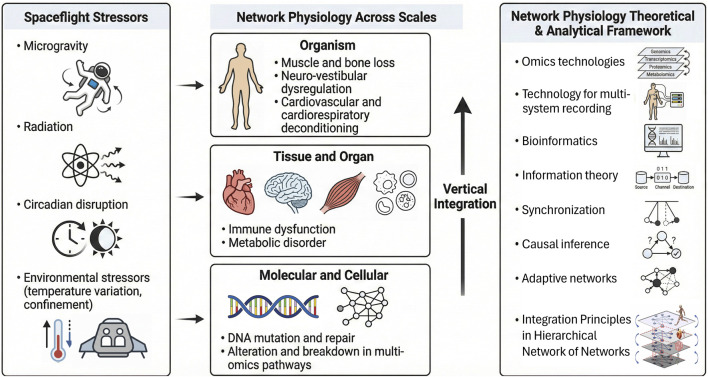
Network Physiology across spatio-temporal scales under spaceflight stressors. Schematic representation of how spaceflight-related stressors—including microgravity, radiation, circadian and sleep disruption, confinement, and psychological strain—perturb physiological organization across spatio-temporal scales in vertical and horizontal integration (left panel). Spaceflight influences DNA mutation and multi-omics pathways at the molecular scale, contributes to immune and metabolic dysfunction at the tissue and organ level, and manifests as muscle and bone loss, neuro-vestibular dysregulation, and cardio-respiratory and cardio-vascular deconditioning at the organism level. Vertical integration refers to the propagation of systems dynamics and network interactions across scales, whereas horizontal integration describes the coordination and network integration among systems at the same organizational level—both may be altered under spaceflight conditions (middle panel). Novel analytical and methodological approaches within the Network Physiology framework can help identify and quantify cross-scale interactions and hierarchical systems-level network of networks integration (right panel).

Recent advances in systems biology, multi-omics, nonlinear dynamics and information theory for multi-variate physiological systems interactions, and computational modeling have enabled deeper exploration of vertical and horizontal integration (Guyton et al., 1955; Guyton et al., 1972; Kitano, 2002; Buchman, 2006; Asada et al., 2016; Moorman et al., 2016). Molecular profiling (genomics, proteomics, metabolomics) provides detailed insight into cellular and tissue-level mechanisms, while imaging and physiological monitoring capture organ-level dynamics. Data-driven network and modeling approaches, including adaptive network theory of dynamical systems provide structural and dynamical representations of inter-organ connectivity and facilitate the study of horizontal integration by quantifying inter-organ interactions (Morandotti et al., 2025; Bartsch et al., 2012; Faes et al., 2014; Lin et al., 2016; Berner et al., 2022; Sawicki et al., 2022). Complementing structural network models, dynamical systems analysis characterizes the temporal evolution, stability, causality, and cross-scale propagation of physiological processes. Within the Network Physiology framework, analytical approaches—such as information-theoretic measures, synchronization analysis, causal inference techniques, and adaptive network modeling—enable quantitative assessment of coupling strength, directionality, time delays, and state-dependent reconfiguration among interacting physiological systems (Figure 3). For example, computational models of the cardiovascular-respiratory system can predict how perturbations in heart rate or lung function propagate through the organism, providing mechanistic insights that are difficult to achieve experimentally or comprehend from reductionist experiments.

Furthermore, integrative experimental platforms, such as organ-on-chip and multi-organ micro-physiological systems, allow researchers to model vertical and horizontal interactions *in vitro*. By combining human cell-derived organoids with microfluidic networks, these platforms recapitulate tissue-specific functions and inter-organ communication, enabling mechanistic studies of drug responses and disease pathophysiology (Wang and Qin, 2023), and providing controlled experimental platforms to investigate hierarchical interactions within a network-of-networks architecture.

### Mathematical modeling of systems dynamics and network interactions from spaceflight data

**FIGURE 4:**
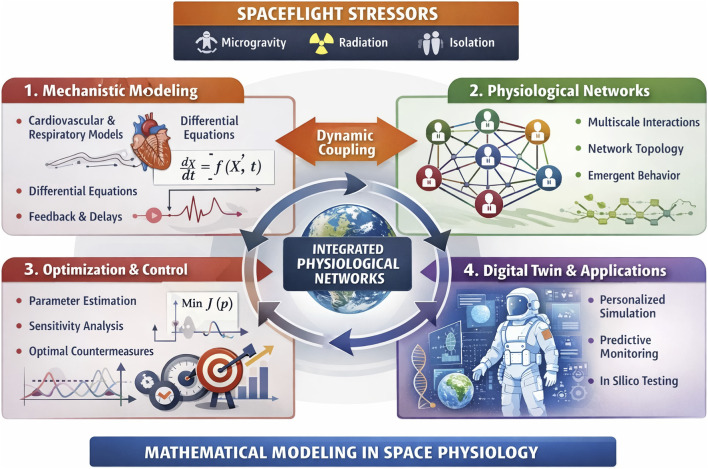
Modeling of physiological systems in spaceflight medicine research. To address multi-system stressors during spaceflight, modern modeling approaches have to extend beyond the complex dynamics and regulatory mechanisms of individual systems, and utilize the Network Physiology framework to account for time-varying adaptive interactions among diverse physiological systems across temporal scales and levels of organization to identify and quantify the mechanistic pathways through which systems hierarchically integrate as a network to generate distinct states and functions at the organism level.

Spaceflight imposes persistent stressors that reshape interactions among physiological systems, altering feedback mechanisms and control structures. These changes give rise to emergent, network-level effects—such as cardiovascular deconditioning, orthostatic intolerance, immune dysfunction, and musculoskeletal wasting—rather than discrete failures of individual systems. Mathematical modeling (Figure 4) offers a rigorous framework to: (i) Describe coupled physiological sub-systems through differential equations, delay dynamics, and control formulations; (ii) Characterize feedback behavior, stability, resilience, and adaptive responses in altered gravity environments; (iii) Forecast whole-system behavior when direct experimentation is limited; (iv) Test and refine countermeasures computationally prior to implementation.

In the context of space physiology, such models are particularly critical because available data are limited, costly to obtain, and subject to ethical constraints.

Mathematical modeling is a valuable tool for analyzing cardiovascular function during spaceflight (Keith Sharp et al., 2013). Previous research has reported how cardiovascular system models support space life sciences research, using post-flight orthostatic intolerance (POI)—a major spaceflight-related concern—as a representative example. POI affects a large proportion of astronauts during post-flight stand tests and manifests as dizziness, fainting, and related symptoms that can impair crew performance and pose safety risks. These effects could be even more severe during missions to the Moon or Mars. For more than a decade, POI has been the primary focus of cardiovascular modeling in bioastronautics, with numerous physiological mechanisms explored. Modeling strategies range from computational models with varying levels of hemodynamic detail to physical models evaluated in parabolic and orbital flight. Mathematical techniques, including parameter sensitivity analysis, can help identify dominant system mechanisms and inform the development of more effective countermeasures. Model validation remains a persistent challenge, underscoring the need for targeted experimental data to improve interpretation of cardiovascular responses. Future work is expected to emphasize subject-specific modeling and integrated physiological analyses, enabling assessment of individual susceptibility to POI and evaluation of cardiovascular effects on musculoskeletal, visual, and cognitive function.

Mathematical modeling, especially when grounded in rigorous control theory and validation methods (Batzel et al., 2007; Batzel et al., 2013), is essential for transforming space physiology from descriptive observation into predictive, network-based, and optimizable science. Additional mathematical models that could be used in Network Physiology include: (i) Mechanistic modeling of cardiovascular, cardio–respiratory, cardio-muscular, and inter-muscular control systems, explicitly addressing nonlinear feedback and transport delays. Such models could become a gold standard for exploration and validation of hypotheses reflecting sub-system interactions, for analyzing system level function, and sensitivity analysis of sub-system parameters; (ii) Model validation, parameter identifiability, and sensitivity analysis, which are critical when working with limited astronaut data; (iii) Optimization of experimental design, ensuring models extract maximal information from minimal measurements; (iv) Application of control theory and adaptive network concepts (stability, regulation, robustness, causality, directionality, synchronization, time-delays, adaptive feedbacks, synergetics and emergence) to physiological systems and their interactions (Rosenblum et al., 1996; Tass et al., 1998; Pikovsky et al., 2001; Ott, 2002; Chen et al., 2006; Xu et al., 2006; Keener and Sneyd, 2009; Haken, 2012; Doyle et al., 2013; Balagué et al., 2020; Zabczyk, 2020; Ivanov, 2021).

Work in this area directly supports the transition from descriptive models to predictive, actionable physiological network models, making it highly relevant for space medicine and long-duration missions. When extended beyond single systems, mechanistic models naturally integrate with Network Physiology, where: (i) nodes represent physiological systems and sub-systems; (ii) network links represent time-varying regulatory coupling, feedback, or information flow; (iii) network structure, dynamics and parameters evolve across spaceflight mission phases (Figure 4).

This hybrid approach bridges network science and control-oriented physiological modeling, enabling both interpretability and prediction. In addition, combination models incorporating mechanistic modelling approaches reflecting known physiological processes and machine learning approaches providing black box input/output modelling using known data when specific physiology is unknown allow for expanded modelling of physiological systems and the use of a variety of data types (Procopio et al., 2023).

### Integration with AI and translational perspectives

Importantly, Network Physiology provides the conceptual physiological structure necessary to guide applications of AI or data-driven modeling. AI and machine learning techniques facilitate pattern discovery, network inference, and prediction in high-dimensional data, and are essential components of the Network Physiology methodological instrumentarium necessary for comprehensive quantification and interpretation in terms of meaningful physiological interactions. To address challenges in Network Physiology, a new generation of physiologically inspired AI and machine learning algorithms are needed, specificity trained to simultaneously respond to both spatial and temporal features of dynamic network and track real-time changes of states and conditions. Hybrid approaches combining mechanistic modeling and machine learning enable computational physiological networks capable of simulating system-wide responses, predicting intervention outcomes, and guiding personalized countermeasures across the entire spaceflight architecture (Ivanov, 2021).

### AI and digital twins: clinical implementation

AI-enabled digital twins have strong potential to advance aerospace medicine through personalized physiological modeling, risk prediction, and decision support, not least in extreme operational environments including spaceflight (Glaessgen and Stargel, 2012; Clément et al., 2015; Hargens and Vico, 2016; Viceconti et al., 2016; Doshi-Velez and Kim, 2017; Topol, 2019; Corral-Acero et al., 2020; Smuck et al., 2021; Nasarian et al., 2024; Elgammal et al., 2025). However, clinical implementation depends on robust validation across heterogeneous and longitudinal data, as well as mission-relevant stressors such as microgravity, hypoxia, circadian disruption, and radiation (Corral-Acero et al., 2020). Operational readiness requires integration with clinical workflows, real-time data from wearable and onboard systems, and compliance with safety-critical regulatory standards (Topol, 2019; Smuck et al., 2021). Transparent and interpretable AI is essential to support effective human–AI teaming and clinical oversight (Doshi-Velez and Kim, 2017; Nasarian et al., 2024). Aerospace systems engineering practices provide a proven framework for addressing these challenges and enabling responsible deployment of digital twins in clinical and aerospace medical settings (Glaessgen and Stargel, 2012) (Figure 4).

Whilst spaceflight is interesting and timely in itself, the integrative framework of Network Physiology positions space as a living laboratory for understanding resilience, adaptation, and aging. Insights gained from extreme environments have been used to inform diagnostics, therapies, and rehabilitation strategies for aging, but also conditions associated with metabolic disease, neurodegeneration, and muscle-skeletal rehabilitation on Earth. Thus, Network Physiology provides an opportunity to reinforce the bidirectional impact of space physiology and terrestrial medicine upon each other.

## Artificial gravity and countermeasures

Artificial gravity, particularly via short-arm human centrifugation, is a promising countermeasure for mitigating multisystem deconditioning during long-duration spaceflight (Lackner and DiZio, 2000; Glaessgen and Stargel, 2012; Clément et al., 2015; Hargens and Vico, 2016; Viceconti et al., 2016; Evans et al., 2018; Elgammal et al., 2025). These systems allow controlled, intermittent hyper gravity exposure within compact and operationally feasible platforms, with precise modulation of gravity dose and timing that is effective (Clément et al., 2015; Laing et al., 2020), but also tolerable (Frett et al., 2020). From an AI and digital twin perspective, artificial gravity provides an effective testbed for personalized countermeasure development, as key physiological responses can be continuously monitored using multi-modal sensing and biomarker data (Hargens and Vico, 2016). Integration of AI-driven digital twins with artificial gravity systems enables closed-loop, individualized optimization of countermeasure protocols based on real-time physiological feedback and mission requirements (Evans et al., 2015; Goswami et al., 2015; Elgammal et al., 2025; Stead et al., 2025). This approach supports precision aerospace medicine and offers a controlled environment for validating digital twin models prior to clinical or mission deployment (Glaessgen and Stargel, 2012).

## The human physiolome on earth and in space

The Human Physiolome (Cartwright, 2016; Ivanov, 2021) represents a comprehensive, data-driven atlas of the dynamic networks of physiological interactions which uniquely define various physiological states, functions and conditions in health and disease that underline life on Earth. The Human Physiolome aims to quantify and catalog the structure and dynamics of physiological systems cross-communications through large-scale multi-modal synchronized recordings and analytical methods, producing blueprint reference network maps (approximately $>10^{7}$ maps (Ivanov, 2021)) that link patterns of systems interaction and network integration to specific states and conditions in health and disease. By capturing billions of data points of physiological signals and coupling dynamics, the Human Physiolome seeks to demonstrate the fundamental principles of network integration and control that govern organism-level functions. Extending this database beyond Earth, by building the Human Physiolome under spaceflight conditions offers opportunities to understand how vertical and horizontal integration among physiological systems is perturbed in space, with implications for astronaut health, countermeasures development, and broader biological insights into adaptability, resilience, systemic organization, and patterns of cross-communication.

## Conclusions and future directions

In summary, hierarchical vertical and horizontal network integration jointly shape physiological function and dysfunction. Vertical integration ensures that molecular and cellular processes coalesce to generate functional organ outputs, while horizontal integration maintains systemic homeostasis through dynamic inter-organ communication. Understanding these dimensions is critical for elucidating the mechanisms underlying health and disease, and for developing interventions that operate across multiple biological scales. Future research leveraging systems biology, computational modeling, and organ-on-chip platforms promises to deepen our mechanistic understanding of physiological integration, paving the way for precision medicine and targeted therapeutics.

Novel computational tools and analytic formalism developed in the field of Network Physiology (Bashan et al., 2012; Ivanov and Bartsch, 2014) have added new rich dimensions to our understanding of physiologic states and functions. The integrative perspective has redefined physiologic states from the point of view of dynamic adaptive networks of systems interactions. This has helped establish the first associations between distinct physiologic states and conditions and both network topology and the temporal characteristics of systems and organ interactions (network links), even when network topology remains unchanged. It has been shown that brain–organ interactions have preferred channels of communication (frequency bands) that are specific for each organ (Bartsch et al., 2015; Rizzo et al., 2020; Rizzo et al., 2023). Recent efforts have focused on brain–heart (Lin et al., 2016; Valenza et al., 2016), cardio-respiratory (Bartsch et al., 2012; Bartsch and Ivanov, 2014; Bartsch et al., 2014), muscle fiber and inter-muscle coordination (Garcia-Retortillo and Ivanov, 2022; Garcia-Retortillo et al., 2023), and cardio-muscular networks (Garcia-Retortillo and Ivanov, 2025) to identify new aspects of coupling dynamics and feedback mechanisms. By developing the theoretical framework necessary to uncover basic principles of (i) integration among diverse physiologic systems (from genomics to organs) that leads to complex physiologic functions at the organism level, and (ii) hierarchical reorganization of physiological networks and their evolution across states and conditions, investigations in the field of Network Physiology provide the building blocks of a first atlas of dynamic organ interactions, the Human Physiolome (Ivanov, 2021).

### Establishing Network Physiology in space: a new frontier in Network Physiology

Spaceflight uniquely exposes humans to microgravity, radiation, isolation, and circadian disruption, challenging the physiological networks that sustain life. Building on developments in the multidisciplinary field of Network Physiology over the last 15 years, there is a clear necessity, vision, and perspective for the development of a new research area, Network Physiology in Space, that would focus on applying network physiology approaches to multi-scale data from space and space-analogue environments to elucidate mechanisms of adaptation, system vulnerability, and recovery. By combining multi-omics integration, advanced physiological monitoring, data-driven computational and network modeling, Network Physiology in Space would study how physiological networks across systems and levels of biological organization reorganize in space, thereby improving our understanding of adverse space effects, including muscle and bone loss, cardio-vascular and cardio-respiratory deconditioning, and immune and neuro-vestibular adaptations. These investigations would also inform diagnostics and therapies relevant to aging, metabolic disorders, and rehabilitation on Earth. Thus, space serves as a model for resilience, advancing both human spaceflight and terrestrial medicine. The importance of research on physiological networks is also evidenced by the recent ASTROAIMED 2025 Consortium recommendations (Goswami et al., 2026b) that advocate for the establishment of Network Physiology in Space as a new research area to investigate human or animal spaceflight data, analogues of spaceflight (e.g., bed rest, dry immersion, isolation) (Pišot et al., 2016; Marusic et al., 2021; Ritzmann et al., 2026), ground-based platforms (e.g., parabolic flights, artificial gravity), or countermeasures such as lower body negative pressure (Goswami et al., 2008; Batzel et al., 2009; Goswami et al., 2019; Goswami, 2023).

Network Physiology in Space will play essential role in addressing fundamental questions related to basic physiology and clinical medicine on Earth and in space, including: *Molecular and cellular networks*: Effects of space stressors on gene expression, epigenetics, proteomics, metabolism, and cell-signaling networks; *Organ and system adaptations*: Network-level coordination among muscle and bone loss, cardiovascular and respiratory changes, immune dysregulation, and neuro-vestibular modulation; *Novel Methodological approaches*: Systems biology, multi-omics integration, network synchronization, adaptive networks, information theory of multivariate signals, causality, time-varying interactions, time delay stability, computational modeling, and biomarker discovery; *Translational applications:* Space-derived diagnostics and interventions for aging, metabolic disease, neurodegeneration, rehabilitation, and resilience*; Multidisciplinary studies*: Integrating physiology and space medicine with bioinformatics, network science, engineering, and comparative analyses across spaceflight and terrestrial health; *Modeling and AI*: Use of mechanistic models and AI (e.g., machine learning, network analysis) to capture complex physiological interactions, infer network dynamics, fill knowledge gaps, and create hybrid predictive models. Integrating AI with mechanistic modeling enables simulation of system-wide responses, personalized countermeasures, optimized treatment strategies, and improved mission planning. Together, these approaches provide a powerful framework for understanding human physiology in extreme environments and for developing safer, more effective countermeasures for long-duration space missions.

Summary of key aspects, novel concepts, and methodology for Networks Physiology in Space Applications:

- Integrated Physiology Across Scales: Physiological function emerges from both vertical integration (molecules → cells → organs) and horizontal integration (dynamic communication between organs), jointly maintaining health and driving disease mechanisms.
- Network Physiology Paradigm: Reframes the body as a dynamic, adaptive network of interactions among systems across levels of organization (from sub-cellular to organs and organism level), where physiological states and functions are defined by network hierarchal organization across systems and sub-systems and by time-varying interactions, not just individual components.
- Organ-System Connectivity: Distinct physiological states are associated with specific network topology, interaction patterns and systems cross-communication channels (e.g., frequency bands) between organs, including brain–heart, cardio-respiratory, cortico-muscular, cardio-muscular, inter-muscular networks.
- Toward the Human Physiolome: Ongoing work is building a comprehensive dynamic atlas of physiological systems interactions, a new-kind of BigData comprising thousands (possibly millions) of blueprint reference maps representing physiological networks and their reorganization with transition across states and conditions in health and disease.
- New Frontier: Network Physiology in Space: Spaceflight introduces extreme stressors (microgravity, radiation, isolation), making it a unique model to study system-wide physiological adaptation, vulnerability, and recovery.
- Research Focus Areas: Multi-scale networks: molecular → cellular → organ systems → organism
- System adaptations: central autonomic and peripheral nervous system, cardio-vascular, cardio-respiratory, musculoskeletal, neuro-vestibular, sleep/circadian systems, immune, metabolic circuitry
- Advanced methods: theory of adaptive dynamic networks, synchronization, time delay stability, cross-frequency coupling, higher-order network interactions, information theory, causality inference, multi-omics, computational modeling, next-generation bio-inspired AI
- Translational impact: aging, neurodegeneration, metabolic disease, rehabilitation, resilience, sleep and circadian disorders
- AI & Systems Modelling: Integration of AI with mechanistic models enables prediction of complex physiological responses based on individual systems dynamics and their adaptive network interactions, personalized interventions, and optimized strategies for both space missions and terrestrial medicine.
- Broader Impact: Space-based research in Network Physiology will advance precision medicine, improve diagnostics and therapies on Earth, and support safe long-duration human spaceflight.

## Data Availability

The original contributions presented in the study are included in the article/supplementary material, further inquiries can be directed to the corresponding authors.

## References

[B1] Abreu R. M. d. Cairo B. Porta A. (2023). On the significance of estimating cardiorespiratory coupling strength in sports medicine. Front. Netw. Physiology 2, 1114733. 10.3389/fnetp.2022.1114733 PMC10013023 36926078

[B2] Amberger J. Bocchini C. A. Scott A. F. Hamosh A. (2009). McKusick's online Mendelian inheritance in man (OMIM®). Nucleic Acids Res. 37 (Suppl. l_1), D793–D796. 10.1093/nar/gkn665 18842627 PMC2686440

[B3] Antonacci Y. Barà C. Zaccaro A. Ferri F. Pernice R. Faes L. (2023). Time-varying information measures: an adaptive estimation of information storage with application to brain-heart interactions. Front. Netw. Physiology 3, 1242505. 10.3389/fnetp.2023.1242505 37920446 PMC10619917

[B4] Armstrong L. E. Bergeron M. F. Lee E. C. Mershon J. E. Armstrong E. M. (2022). Overtraining syndrome as a complex systems phenomenon. Front. Netw. Physiology 1, 794392. 10.3389/fnetp.2021.794392 36925581 PMC10013019

[B5] Asada T. Aoki Y. Sugiyama T. Yamamoto M. Ishii T. Kitsuta Y. (2016). Organ system network disruption in nonsurvivors of critically ill patients. Crit. Care Med. 44 (1), 83–90. 10.1097/ccm.0000000000001354 26496455

[B6] Bailey D. M. Blottner D. Gunga H.-C. Schneider S. Wotring V. Baatout S. (2025). Integrative focus on the space exposome-integrome: physiological challenges and practical limits of countermeasures beyond low Earth orbit. Npj Microgravity 11 (1), 82. 10.1038/s41526-025-00537-1 41266377 PMC12635128

[B7] Balagué N. Hristovski R. Almarcha M. d.C. Garcia-Retortillo S. Ivanov P. C. (2020). Network physiology of exercise: vision and perspectives. Front. Physiology 11, 611550. 10.3389/fphys.2020.611550 33362584 PMC7759565

[B8] Balagué N. Garcia-Retortillo S. Hristovski R. Ivanov P. C. (2022a). “From exercise physiology to network physiology of exercise,” in Exercise physiology. Editors Ferraz R. Pereira Neiva H. Marinho D. A. A. Teixeira J. E. Forte P. M. Branquinho L. (London: IntechOpen).

[B9] Balagué N. Hristovski R. Almarcha M. Garcia-Retortillo S. Ivanov P. C. (2022b). Network physiology of exercise: beyond molecular and omics perspectives. Sports Med. - Open 8 (1), 119. 10.1186/s40798-022-00512-0 36138329 PMC9500136

[B10] Barabási A.-L. Gulbahce N. Loscalzo J. (2011). Network medicine: a network-based approach to human disease. Nat. Rev. Genet. 12 (1), 56–68. 10.1038/nrg2918 21164525 PMC3140052

[B11] Bartsch R. P. Ivanov P. C. (2014). Coexisting forms of coupling and phase-transitions in physiological networks. Cham: Springer International Publishing, 270–287.

[B12] Bartsch R. P. Schumann A. Y. Kantelhardt J. W. Penzel T. Ivanov P. C. (2012). Phase transitions in physiologic coupling. Proc. Natl. Acad. Sci. 109(26)**,** 10181–10186. 10.1073/pnas.1204568109 22691492 PMC3387128

[B13] Bartsch R. P. Liu K. K. Ma Q. D. Ivanov P. C. (2014). “Three independent forms of cardio-respiratory coupling: transitions across sleep stages,” in *Computing in cardiology 2014* , 781–784. PMC4319215 25664348

[B14] Bartsch R. P. Liu K. K. L. Bashan A. Ivanov P. C. (2015). Network physiology: how organ systems dynamically interact. PLOS ONE 10 (11), e0142143. 10.1371/journal.pone.0142143 26555073 PMC4640580

[B15] Bashan A. Bartsch R. P. Kantelhardt J. W. Havlin S. Ivanov P. C. (2012). Network physiology reveals relations between network topology and physiological function. Nat. Commun. 3 (1), 702. 10.1038/ncomms1705 22426223 PMC3518900

[B16] Batzel J. J. Kappel F. Schneditz D. Tran H. T. (2007). Cardiovascular and respiratory systems: modeling, analysis, and control. Philadelphia, PA, United States: Society for Industrial and Applied Mathematics.

[B17] Batzel J. J. Goswami N. Lackner H. K. Roessler A. Bachar M. Kappel F. (2009). Patterns of cardiovascular control during repeated tests of orthostatic loading. Cardiovasc. Eng. 9 (4), 134–143. 10.1007/s10558-009-9086-z 19813090

[B18] Batzel J. J. Bachar M. Kappel F. (2013). Mathematical modeling and validation in physiology. Berlin Heidelberg: Springer.

[B19] Berner R. Sawicki J. Thiele M. Löser T. Schöll E. (2022). Critical parameters in dynamic network modeling of sepsis. Front. Netw. Physiology 2, 904480. 10.3389/fnetp.2022.904480 36926088 PMC10012967

[B20] Bers D. M. (2002). Cardiac excitation–contraction coupling. Nature 415 (6868), 198–205. 10.1038/415198a 11805843

[B21] Borovkova E. I. Prokhorov M. D. Kiselev A. R. Hramkov A. N. Mironov S. A. Agaltsov M. V. (2022). Directional couplings between the respiration and parasympathetic control of the heart rate during sleep and wakefulness in healthy subjects at different ages. Front. Netw. Physiology 2, 942700. 10.3389/fnetp.2022.942700 36926072 PMC10013057

[B22] Buchman T. G. (2006). “Physiologic failure: multiple organ dysfunction syndrome,” in Complex systems science in biomedicine. Editors Deisboeck T. S. Kresh J. Y. (Boston, MA: Springer US), 631–640.

[B23] Carretero O. A. Oparil S. (2000). Essential hypertension. Circulation 101(3)**,** 329–335. 10.1161/01.CIR.101.3.329 10645931

[B24] Cartwright J. (2016). Revealing the network within. Phys. World. Available online at: https://physicsworld.com/a/revealing-the-network-within/ (Accessed February 9, 2026).

[B25] Charmandari E. Tsigos C. Chrousos G. (2005). Endocrinology of the stress response. Annu. Rev. Physiology 67, 259–284. 10.1146/annurev.physiol.67.040403.120816 15709959

[B26] Chen Z. Hu K. Stanley H. E. Novak V. Ivanov P. C. (2006). Cross-correlation of instantaneous phase increments in pressure-flow fluctuations: applications to cerebral autoregulation. Phys. Rev. E 73 (3), 031915. 10.1103/PhysRevE.73.031915 16605566 PMC2140229

[B27] Chen B. Ciria L. F. Hu C. Ivanov P. C. (2022). Ensemble of coupling forms and networks among brain rhythms as function of states and cognition. Commun. Biol. 5 (1), 82. 10.1038/s42003-022-03017-4 35064204 PMC8782865

[B28] Clément G. R. Bukley A. P. Paloski W. H. (2015). Artificial gravity as a countermeasure for mitigating physiological deconditioning during long-duration space missions. Front. Syst. Neurosci. 9, 92. 10.3389/fnsys.2015.00092 26136665 PMC4470275

[B29] Corral-Acero J. Margara F. Marciniak M. Rodero C. Loncaric F. Feng Y. (2020). The ‘Digital Twin’ to enable the vision of precision cardiology. Eur. Heart J. 41 (48), 4556–4564. 10.1093/eurheartj/ehaa159 32128588 PMC7774470

[B30] Cryan J. F. Dinan T. G. (2012). Mind-altering microorganisms: the impact of the gut microbiota on brain and behaviour. Nat. Rev. Neurosci. 13 (10), 701–712. 10.1038/nrn3346 22968153

[B31] DeFronzo R. A. Ferrannini E. Groop L. Henry R. R. Herman W. H. Holst J. J. (2015). Type 2 diabetes mellitus. Nat. Rev. Dis. Prim. 1 (1), 15019. 10.1038/nrdp.2015.19 27189025

[B32] Dervić E. Ledebur K. Thurner S. Klimek P. (2025). Comorbidity networks from population-wide health data: aggregated data of 8.9M hospital patients (1997–2014). Sci. Data 12 (1), 215. 10.1038/s41597-025-04508-9 39910117 PMC11799221

[B33] Doshi-Velez F. Kim B. (2017). Towards a rigorous science of interpretable machine learning. arXiv Preprint arXiv:1702.08608.

[B34] Doyle J. C. Francis B. A. Tannenbaum A. R. (2013). Feedback control theory. Cour. Corp.

[B35] Elgammal Z. Albrijawi M. T. Alhajj R. (2025). Digital twins in healthcare: a review of AI-powered practical applications across health domains. J. Big Data 12 (1), 234. 10.1186/s40537-025-01280-w

[B36] Evans J. M. Ribeiro L. C. Moore F. B. Wang S. Zhang Q. Kostas V. (2015). Hypovolemic men and women regulate blood pressure differently following exposure to artificial gravity. Eur. J. Appl. Physiology 115 (12), 2631–2640. 10.1007/s00421-015-3261-2 26438067

[B37] Evans J. M. Knapp C. F. Goswami N. (2018). Artificial gravity as a countermeasure to the cardiovascular deconditioning of spaceflight: gender perspectives. Front. Physiology 9, 716. 10.3389/fphys.2018.00716 30034341 PMC6043777

[B38] Faes L. Nollo G. Jurysta F. Marinazzo D. (2014). Information dynamics of brain–heart physiological networks during sleep. New J. Phys. 16 (10), 105005. 10.1088/1367-2630/16/10/105005

[B39] Frett T. Green D. A. Mulder E. Noppe A. Arz M. Pustowalow W. (2020). Tolerability of daily intermittent or continuous short-arm centrifugation during 60-day 6o head down bed rest (AGBRESA study). PLOS ONE 15 (9), e0239228. 10.1371/journal.pone.0239228 32946482 PMC7500599

[B40] Garcia-Retortillo S. Ivanov P. C. (2022). Inter-muscular networks of synchronous muscle fiber activation. Front. Netw. Physiology 2, 2. 10.3389/fnetp.2022.1059793 36926057 PMC10012969

[B41] Garcia-Retortillo S. Ivanov P. C. (2025). Dynamics of cardio-muscular networks in exercise and fatigue. J. Physiology 603 (18), 5121–5147. 10.1113/JP286963 39392864

[B42] Garcia-Retortillo S. Romero-Gómez C. Ivanov P. C. (2023). Network of muscle fibers activation facilitates inter-muscular coordination, adapts to fatigue and reflects muscle function. Commun. Biol. 6 (1), 891. 10.1038/s42003-023-05204-3 37648791 PMC10468525

[B43] Garcia-Retortillo S. Abenza Ó. Thiamwong L. Xie R. Gordon M. Ivanov P. C. (2025a). Case report: network physiology markers of inter-muscular interactions indicate reversal of age decline with exercise training. Front. Netw. Physiology 5, 1686723. 10.3389/fnetp.2025.1686723 41281900 PMC12635726

[B44] Garcia-Retortillo S. Abenza Ó. Vasileva F. Balagué N. Hristovski R. Wells A. (2025b). Age-related breakdown in networks of inter-muscular coordination. GeroScience 47 (2), 1615–1639. 10.1007/s11357-024-01331-9 39287879 PMC11978574

[B45] Glaessgen E. Stargel D. (2012). “The digital twin paradigm for future NASA and US air force vehicles,” in 53rd AIAA/ASME/ASCE/AHS/ASC structures, structural dynamics and materials conference 20th AIAA/ASME/AHS adaptive structures conference 14th AIAA, 1818.

[B46] Goswami N. (2023). Compensatory hemodynamic changes in response to central hypovolemia in humans: lower body negative pressure: updates and perspectives. J. Muscle Res. Cell Motil. 44 (2), 89–94. 10.1007/s10974-022-09635-z 36380185 PMC10329599

[B47] Goswami N. Loeppky J. A. Hinghofer-Szalkay H. (2008). LBNP: past protocols and technical considerations for experimental design. Aviat. Space, Environ. Med. 79 (5), 459–471. 10.3357/ASEM.2161.2008 18500042

[B48] Goswami N. Roma P. G. De Boever P. Clément G. Hargens A. R. Loeppky J. A. (2012). Using the moon as a high-fidelity analogue environment to study biological and behavioral effects of long-duration space exploration. Planet. Space Sci. 74 (1), 111–120. 10.1016/j.pss.2012.07.030

[B49] Goswami N. Batzel J. J. Clément G. Stein T. P. Hargens A. R. Sharp M. K. (2013). Maximizing information from space data resources: a case for expanding integration across research disciplines. Eur. J. Appl. Physiology 113 (7), 1645–1654. 10.1007/s00421-012-2507-5 23073848

[B50] Goswami N. Evans J. Schneider S. von der Wiesche M. Mulder E. Rössler A. (2015). Effects of individualized centrifugation training on orthostatic tolerance in men and women. PLOS ONE 10 (5), e0125780. 10.1371/journal.pone.0125780 26020542 PMC4447337

[B51] Goswami N. Blaber A. P. Hinghofer-Szalkay H. Convertino V. A. (2019). Lower body negative pressure: physiological effects, applications, and implementation. Physiol. Rev. 99 (1), 807–851. 10.1152/physrev.00006.2018 30540225

[B52] Goswami N. White O. Blaber A. Evans J. van Loon J. J. W. A. Clement G. (2021). Human physiology adaptation to altered gravity environments. Acta Astronaut. 189, 216–221. 10.1016/j.actaastro.2021.08.023

[B53] Goswami N. Blaber A. P. Valenti G. Hinghofer-Szalkay H. Evans J. Bailey D. M. (2026a). Gravity, microgravity, and artificial gravity: physiological effects, implementation, and applications. Physiol. Rev. 106 (2), 751–840. 10.1152/physrev.00055.2024 41021767

[B54] Goswami N. Fredriksen P. M. Al Suwaidi H. Soares N. Al-Falasi S. Bin Nashoouq M. Y. (2026b). ASTROAIMED consensus statement: advancing space omics, AI integration and network physiology for precision health of astronauts in space and patients on Earth. Under Rev.

[B55] Green D. A. (2024). SpaceX must share astronaut health data to boost space biomedicine. Nature 634 (8035), 782. 10.1038/d41586-024-03438-7 39438747

[B56] Günther M. Kantelhardt J. W. Bartsch R. P. (2022). The reconstruction of causal networks in physiology. Front. Netw. Physiology 2, 893743. 10.3389/fnetp.2022.893743 36926108 PMC10013035

[B57] Guyton A. C. Lindsey A. W. Kaufmann B. N. (1955). Effect of mean circulatory filling pressure and other peripheral circulatory factors on cardiac output. Am. J. Physiology-Legacy Content 180 (3), 463–468. 10.1152/ajplegacy.1955.180.3.463 14376522

[B58] Guyton A. C. Coleman T. G. Granger H. J. (1972). Circulation: overall regulation. Annu. Rev. Physiology 34, 13–44. 10.1146/annurev.ph.34.030172.000305 4334846

[B59] Haken H. (2012). Advanced synergetics: instability hierarchies of self-organizing systems and devices. Springer Science & Business Media.

[B60] Hall J. E. (2016). Guyton and hall textbook of medical physiology, Jordanian edition. E-Book. Elsevier Health Sciences.

[B61] Hardie D. G. Ross F. A. Hawley S. A. (2012). AMPK: a nutrient and energy sensor that maintains energy homeostasis. Nat. Rev. Mol. Cell Biol. 13 (4), 251–262. 10.1038/nrm3311 22436748 PMC5726489

[B62] Hargens A. R. Vico L. (2016). Long-duration bed rest as an analog to microgravity. J. Appl. Physiology 120 (8), 891–903. 10.1152/japplphysiol.00935.2015 26893033

[B63] Haug N. Sorger J. Gisinger T. Gyimesi M. Kautzky-Willer A. Thurner S. (2021). Decompression of multimorbidity along the disease trajectories of diabetes mellitus patients. Front. Physiology 11, 612604. 10.3389/fphys.2020.612604 33469431 PMC7813935

[B64] Healy K. L. Morris A. R. Liu A. C. (2021). Circadian synchrony: sleep, nutrition, and physical activity. Front. Netw. Physiology 1, 732243. 10.3389/fnetp.2021.732243 35156088 PMC8830366

[B65] Ivanov P. C. (2021). The new field of network physiology: building the human physiolome. Front. Netw. Physiology 1, 711778. 10.3389/fnetp.2021.711778 36925582 PMC10013018

[B66] Ivanov P. C. Bartsch R. P. (2014). “Network physiology: mapping interactions between networks of physiologic networks,” in Networks of networks: the last frontier of complexity. Editors D'Agostino G. Scala A. (Cham: Springer International Publishing), 203–222.

[B67] Ivanov P. C. Bartsch R. P. (2025). Future of sleep medicine: novel insights on sleep regulation from network physiology (part II). Sleep. Med. Clin. 20 (1), 149–164. 10.1016/j.jsmc.2024.10.013 39894595

[B68] Ivanov P. C. Bunde A. Amaral L. A. N. Havlin S. Fritsch-Yelle J. Baevsky R. M. (1999). Sleep-wake differences in scaling behavior of the human heartbeat: analysis of terrestrial and long-term space flight data. Europhys. Lett. 48 (5), 594–600. 10.1209/epl/i1999-00525-0 11542917

[B69] Ivanov P. C. Liu K. K. L. Bartsch R. P. (2016). Focus on the emerging new fields of network physiology and network medicine. New J. Phys. 18 (10), 100201. 10.1088/1367-2630/18/10/100201 30881198 PMC6415921

[B70] Ivanov P. C. Liu K. K. L. Lin A. Bartsch R. P. (2017). Network physiology: from neural plasticity to organ network interactions. Springer International Publishing, 145–165.

[B71] Ivanov P. C. Wang J. W. J. L. Zhang X. Chen B. (2021). “The new frontier of network physiology: emerging physiologic states in health and disease from integrated organ network interactions,” in 2019-20 MATRIX annals. Editors de Gier J. Praeger C. E. Tao T. (Cham: Springer International Publishing), 237–254.

[B72] Joyner M. J. (2011). Physiology: alone at the bottom, alone at the top. J. Physiology 589 (5), 1005. 10.1113/jphysiol.2010.203893 21486821 PMC3060580

[B73] Keener J. Sneyd J. (2009). Mathematical physiology 1: cellular physiology. New York, NY, USA: Springer.

[B74] Keith Sharp M. Batzel J. J. Montani J.-P. (2013). Space physiology IV: mathematical modeling of the cardiovascular system in space exploration. Eur. J. Appl. Physiology 113 (8), 1919–1937. 10.1007/s00421-013-2623-x 23539439

[B75] Kerkman J. N. Daffertshofer A. Gollo L. L. Breakspear M. Boonstra T. W. (2018). Network structure of the human musculoskeletal system shapes neural interactions on multiple time scales. Sci. Adv. 4(6)**,** eaat0497. 10.1126/sciadv.aat0497 29963631 PMC6021138

[B76] Kirouac D. C. Ito C. Csaszar E. Roch A. Yu M. Sykes E. A. (2010). Dynamic interaction networks in a hierarchically organized tissue. Mol. Syst. Biol. 6 (1), MSB201071. 10.1038/msb.2010.71 20924352 PMC2990637

[B77] Kitano H. (2002). Systems biology: a brief overview. Science 295(5560)**,** 1662–1664. 10.1126/science.1069492 11872829

[B78] Köhler S. Bauer S. Horn D. Robinson P. N. (2008). Walking the interactome for prioritization of candidate disease genes. Am. J. Hum. Genet. 82 (4), 949–958. 10.1016/j.ajhg.2008.02.013 18371930 PMC2427257

[B79] Lackner J. R. DiZio P. (2000). Artificial gravity as a countermeasure in long-duration space flight. J. Neurosci. Res. 62 (2), 169–176. 10.1002/1097-4547(20001015)62:2<169::AID-JNR2>3.0.CO;2-B 11020210

[B80] Lage K. Hansen N. T. Karlberg E. O. Eklund A. C. Roque F. S. Donahoe P. K. (2008). A large-scale analysis of tissue-specific pathology and gene expression of human disease genes and complexes. Proc. Natl. Acad. Sci. 105(52)**,** 20870–20875. 10.1073/pnas.0810772105 19104045 PMC2606902

[B81] Lage K. Møllgård K. Greenway S. Wakimoto H. Gorham J. M. Workman C. T. (2010). Dissecting spatio‐temporal protein networks driving human heart development and related disorders. Mol. Syst. Biol. 6 (1), MSB201036. 10.1038/msb.2010.36 20571530 PMC2913399

[B82] Laing C. Green D. A. Mulder E. Hinghofer-Szalkay H. Blaber A. P. Rittweger J. (2020). Effect of novel short-arm human centrifugation-induced gravitational gradients upon cardiovascular responses, cerebral perfusion and g-tolerance. J. Physiology 598 (19), 4237–4249. 10.1113/JP273615 32715482 PMC7589294

[B83] Lee D.-S. Park J. Kay K. A. Christakis N. A. Oltvai Z. N. Barabási A.-L. (2008). The implications of human metabolic network topology for disease comorbidity. Proc. Natl. Acad. Sci. 105(29)**,** 9880–9885. 10.1073/pnas.0802208105 18599447 PMC2481357

[B84] Lehnertz K. Bröhl T. Rings T. (2020). The human organism as an integrated interaction network: recent conceptual and methodological challenges. Front. Physiology 11, 598694. 10.3389/fphys.2020.598694 33408639 PMC7779628

[B85] Lin A. Liu K. K. L. Bartsch R. P. Ivanov P. C. (2016). Delay-correlation landscape reveals characteristic time delays of brain rhythms and heart interactions. Philosophical Trans. R. Soc. A Math. Phys. Eng. Sci. 374 (2067), 20150182. 10.1098/rsta.2015.0182 27044991 PMC4822443

[B86] Lin A. Liu K. K. L. Bartsch R. P. Ivanov P. C. (2020). Dynamic network interactions among distinct brain rhythms as a hallmark of physiologic state and function. Commun. Biol. 3 (1), 197. 10.1038/s42003-020-0878-4 32341420 PMC7184753

[B87] Liu K. K. L. Bartsch R. P. Lin A. Mantegna R. N. Ivanov P. C. (2015a). Plasticity of brain wave network interactions and evolution across physiologic states. Front. Neural Circuits 9, 62. 10.3389/fncir.2015.00062 26578891 PMC4620446

[B88] Liu K. K. L. Bartsch R. P. Ma Q. D. Y. Ivanov P. C. (2015b). Major component analysis of dynamic networks of physiologic organ interactions. J. Phys. Conf. Ser. 640 (1), 012013. 10.1088/1742-6596/640/1/012013 30174717 PMC6119077

[B89] Marusic U. Narici M. Simunic B. Pisot R. Ritzmann R. (2021). Nonuniform loss of muscle strength and atrophy during bed rest: a systematic review. J. Appl. Physiology 131 (1), 194–206. 10.1152/japplphysiol.00363.2020 33703945 PMC8325614

[B90] Mason C. E. Green J. Adamopoulos K. I. Afshin E. E. Baechle J. J. Basner M. (2024). A second space age spanning omics, platforms and medicine across orbits. Nature 632 (8027), 995–1008. 10.1038/s41586-024-07586-8 38862027 PMC12366838

[B91] Milo R. Shen-Orr S. Itzkovitz S. Kashtan N. Chklovskii D. Alon U. (2002). Network motifs: simple building blocks of complex networks. Science 298(5594)**,** 824–827. 10.1126/science.298.5594.824 12399590

[B92] Moorman J. R. Lake D. E. Ivanov P. C. (2016). Early detection of sepsis—A role for network physiology? Crit. Care Med. 44 (5), e312–e313. 10.1097/ccm.0000000000001548 27083036

[B93] Morandotti C. Rigny L. Williams T. B. Badariotti J. I. Miller-Dicks M. Bhogal A. S. (2025). Non-invasive assessment of integrated cardiorespiratory network dynamics after physiological stress in humans. J. Physiology. 10.1113/JP288939 40623438

[B94] Nasarian E. Alizadehsani R. Acharya U. R. Tsui K.-L. (2024). Designing interpretable ML system to enhance trust in healthcare: a systematic review to proposed responsible clinician-AI-collaboration framework. Inf. Fusion 108, 102412. 10.1016/j.inffus.2024.102412

[B95] Oti M. Snel B. Huynen M. A. Brunner H. G. (2006). Predicting disease genes using protein–protein interactions. J. Med. Genet. 43 (8), 691–698. 10.1136/jmg.2006.041376 16611749 PMC2564594

[B96] Ott E. (2002). Chaos in dynamical systems. Cambridge: Cambridge University Press.

[B97] Park J. Lee D. S. Christakis N. A. Barabási A. L. (2009). The impact of cellular networks on disease comorbidity. Mol. Syst. Biol. 5 (1), MSB200916. 10.1038/msb.2009.16 19357641 PMC2683720

[B98] Pikovsky A. Rosenblum M. Kurths J. (2001). Synchronization: a universal concept in nonlinear sciences. Cambridge: Cambridge University Press.

[B99] Pišot R. Marusic U. Biolo G. Mazzucco S. Lazzer S. Grassi B. (2016). Greater loss in muscle mass and function but smaller metabolic alterations in older compared with younger men following 2 wk of bed rest and recovery. J. Appl. Physiology 120 (8), 922–929. 10.1152/japplphysiol.00858.2015 26823343

[B100] Procopio A. Cesarelli G. Donisi L. Merola A. Amato F. Cosentino C. (2023). Combined mechanistic modeling and machine-learning approaches in systems biology – a systematic literature review. Comput. Methods Programs Biomed. 240, 107681. 10.1016/j.cmpb.2023.107681 37385142

[B101] Reverter A. Ingham A. Dalrymple B. P. (2008). Mining tissue specificity, gene connectivity and disease association to reveal a set of genes that modify the action of disease causing genes. BioData Min. 1 (1), 8. 10.1186/1756-0381-1-8 18822114 PMC2556670

[B102] Ritzmann R. Centner C. Hughes L. Waldvogel J. Marusic U. (2026). Neuromotor changes in postural control following bed rest. J. Physiology 604 (2), 761–782. 10.1113/JP285668 40237347 PMC12810218

[B103] Rizzo R. Zhang X. Wang J. W. J. L. Lombardi F. Ivanov P. C. (2020). Network physiology of cortico–muscular interactions. Front. Physiology 11, 558070. 10.3389/fphys.2020.558070 33324233 PMC7726198

[B104] Rizzo R. Garcia-Retortillo S. Ivanov P. C. (2022). Dynamic networks of physiologic interactions of brain waves and rhythms in muscle activity. Hum. Mov. Sci. 84, 102971. 10.1016/j.humov.2022.102971 35724499

[B105] Rizzo R. Wang J. W. J. L. DePold Hohler A. Holsapple J. W. Vaou O. E. Ivanov P. C. (2023). Dynamic networks of cortico-muscular interactions in sleep and neurodegenerative disorders. Front. Netw. Physiology 3, 1168677. 10.3389/fnetp.2023.1168677 37744179 PMC10512188

[B106] Roeder L. Boonstra T. W. Kerr G. K. (2020). Corticomuscular control of walking in older people and people with Parkinson’s disease. Sci. Rep. 10 (1), 2980. 10.1038/s41598-020-59810-w 32076045 PMC7031238

[B107] Roeder L. Breakspear M. Kerr G. K. Boonstra T. W. (2024). Dynamics of brain-muscle networks reveal effects of age and somatosensory function on gait. iScience 27 (3), 109162. 10.1016/j.isci.2024.109162 38414847 PMC10897916

[B108] Romero-Ortuño R. Martínez-Velilla N. Sutton R. Ungar A. Fedorowski A. Galvin R. (2021). Network physiology in aging and frailty: the grand challenge of physiological reserve in older adults. Front. Netw. Physiology 1, 712430. 10.3389/fnetp.2021.712430 36925570 PMC10012993

[B109] Rosenblum M. G. Pikovsky A. S. Kurths J. (1996). Phase synchronization of chaotic oscillators. Phys. Rev. Lett. 76 (11), 1804–1807. 10.1103/PhysRevLett.76.1804 10060525

[B110] Sawicki J. Berner R. Löser T. Schöll E. (2022). Modeling tumor disease and sepsis by networks of adaptively coupled phase oscillators. Front. Netw. Physiology 1, 1. 10.3389/fnetp.2021.730385 36925568 PMC10013027

[B111] Scott J. P. R. Weber T. Green D. A. (2020). Editorial: optimization of exercise countermeasures for human space flight—lessons from terrestrial physiology and operational implementation. Front. Physiology 10, 1567. 10.3389/fphys.2019.01567 31998142 PMC6965165

[B112] Scott J. P. R. Kramer A. Petersen N. Green D. A. (2021). The role of long-term head-down bed rest in understanding inter-individual variation in response to the spaceflight environment: a perspective review. Front. Physiology 12, 614619. 10.3389/fphys.2021.614619 33643065 PMC7904881

[B113] Siddiqui R. Qaisar R. Goswami N. Khan N. A. Elmoselhi A. (2021). Effect of microgravity environment on gut microbiome and angiogenesis. Life 11 (10), 1008. 10.3390/life11101008 34685381 PMC8541308

[B114] Smuck M. Odonkor C. A. Wilt J. K. Schmidt N. Swiernik M. A. (2021). The emerging clinical role of wearables: factors for successful implementation in healthcare. Npj Digit. Med. 4 (1), 45. 10.1038/s41746-021-00418-3 33692479 PMC7946921

[B115] Song C. Havlin S. Makse H. A. (2005). Self-similarity of complex networks. Nature 433 (7024), 392–395. 10.1038/nature03248 15674285

[B116] Stead T. Blaber A. P. Divsalar D. N. Xu D. Tavakolian K. Evans J. (2025). Repeatability of artificial gravity tolerance times. Front. Physiology 16, 1464028. 10.3389/fphys.2025.1464028 40386622 PMC12082469

[B117] Stuart J. M. Segal E. Koller D. Kim S. K. (2003). A gene-coexpression network for global discovery of conserved genetic modules. Science 302(5643)**,** 249–255. 10.1126/science.1087447 12934013

[B118] Takayasu L. Suda W. Takanashi K. Iioka E. Kurokawa R. Shindo C. (2017). Circadian oscillations of microbial and functional composition in the human salivary microbiome. DNA Res. 24 (3), 261–270. 10.1093/dnares/dsx001 28338745 PMC5499806

[B119] Taniguchi C. M. Emanuelli B. Kahn C. R. (2006). Critical nodes in signalling pathways: insights into insulin action. Nat. Rev. Mol. Cell Biol. 7 (2), 85–96. 10.1038/nrm1837 16493415

[B120] Tass P. Rosenblum M. G. Weule J. Kurths J. Pikovsky A. Volkmann J. (1998). Detection of n:m phase locking from noisy data: application to magnetoencephalography. Phys. Rev. Lett. 81 (15), 3291–3294. 10.1103/PhysRevLett.81.3291

[B121] Tilg H. Moschen A. R. (2010). Evolution of inflammation in nonalcoholic fatty liver disease: the multiple parallel hits hypothesis. Hepatology 52 (5), 1836–1846. 10.1002/hep.24001 21038418

[B122] Topol E. J. (2019). High-performance medicine: the convergence of human and artificial intelligence. Nat. Med. 25 (1), 44–56. 10.1038/s41591-018-0300-7 30617339

[B123] Tubbs A. S. Fernandez F.-X. Grandner M. A. Perlis M. L. Klerman E. B. (2022). The mind after midnight: nocturnal wakefulness, behavioral dysregulation, and psychopathology. Front. Netw. Physiology 1, 830338. 10.3389/fnetp.2021.830338 35538929 PMC9083440

[B124] Valenza G. Toschi N. Barbieri R. (2016). Uncovering brain–heart information through advanced signal and image processing. Philosophical Trans. R. Soc. A Math. Phys. Eng. Sci. 374 (2067), 20160020. 10.1098/rsta.2016.0020 27044995 PMC4822450

[B125] Valenza G. Matić Z. Catrambone V. (2025). The brain–heart axis: integrative cooperation of neural, mechanical and biochemical pathways. Nat. Rev. Cardiol. 22 (8), 537–550. 10.1038/s41569-025-01140-3 40033035

[B126] Viceconti M. Henney A. Morley-Fletcher E. (2016). *In silico* clinical trials: how computer simulation will transform the biomedical industry. Int. J. Clin. Trials 3 (2), 37–46. 10.18203/2349-3259.ijct20161408

[B127] Voliotis M. Garner K. L. Alobaid H. Tsaneva-Atanasova K. McArdle C. A. (2018). “Exploring dynamics and noise in gonadotropin-releasing hormone (GnRH) signaling,” in Computational cell biology: methods and protocols. Editors von Stechow L. Santos Delgado A. (New York, NY: Springer), 405–429. 10.1007/978-1-4939-8618-7_19 30421415

[B128] Wang Y. Qin J. (2023). Advances in human organoids-on-chips in biomedical research. Life Med. 2 (1), lnad007. 10.1093/lifemedi/lnad007 39872958 PMC11749282

[B129] Xu L. Chen Z. Hu K. Stanley H. E. Ivanov P. C. (2006). Spurious detection of phase synchronization in coupled nonlinear oscillators. Phys. Rev. E 73 (6), 065201. 10.1103/PhysRevE.73.065201 16906897

[B130] Zabczyk J. (2020). Mathematical control theory. Springer.

